# Immunostimulatory effects on B cells by the chemotherapeutic drug Doxorubicin

**DOI:** 10.1186/1479-5876-9-S2-P17

**Published:** 2011-11-23

**Authors:** Johan M Kinn, Ali Zirakzadeh, Jin Hu, Ola Winqvist

**Affiliations:** 1 Dept. of Medicine, Translational Immunology Unit, Karolinska Institutet, Stockholm, Sweden

## Introduction

The efficacy of chemotherapeutic drugs has traditionally been evaluated by their ability to exhibit a direct cytotoxic effect on malignant cells. Recently, it has been reported that certain of these drugs can also enhance the antitumor efficacy and immunotherapy response through their capacity to modulate innate and adaptive immunity [1]. Our research group has earlier utilized a treatment protocol based on the principle that tumor reactive T cells can be isolated from sentinel lymph nodes, expanded in vitro and then be given back to the patient as an autologous transfusion [2]. B cells are the most abundant APC in lymph nodes, and it is therefore of interest to study the effects of chemotherapeutic drugs to B cell antigen presenting function.

## Materials and Methods

Human CD19^+^ B cells were preincubated with chemotherapeutic drugs over night. Next day these cells were used in the FASCIA assay [3], to evaluate the proliferative response of autologous CD4^+^ T cells. The proliferative response was analyzed with FACS, by identifying lymphoblasts as CD3^+^/CD4^+^/CD45RO^+^/HLA-DR^+^ cells. Further analysis of several B-cell surface markers revealed that the co-stimulatory molecule CD86 was upregulated by some drugs. The mRNA expression of CD86 and associated regulatory genes were also analyzed by QT-PCR.

## Results

Incubation of B cells with Doxorubicin increased the proliferative response in the functional FASCIA assay, while several other drugs tested caused suppression. Further experiments revealed this effect to be due to an increased expression of the co-stimulatory molecule CD86, and inhibition experiments with the addition of anti-CD86 antibody reversed the observed finding.

## Conclusions

We have demonstrated that Doxorubicin treatment of human CD19^+^ B cells leads to an increased expression of CD86, and increased antigen presenting function in the FASCIA assay. Gained knowledge of the immunomodulatory properties of chemotherapeutic drugs will permit the development of drug dose and time schedules which allow for recovery of immune function, and may possibly lead to augmented anti-tumor responses.
